# Public Mental Health Approaches to Online Radicalisation: An Empty Systematic Review

**DOI:** 10.3390/ijerph20166586

**Published:** 2023-08-16

**Authors:** Rabya Mughal, Valerie DeMarinis, Maria Nordendahl, Hassan Lone, Veronica Phillips, Eolene Boyd-MacMillan

**Affiliations:** 1 Department of Psychiatry, Cambridge Public Health, Herchel Smith Building, School of Clinical Medicine, University of Cambridge, Cambridge CB2 0SZ, UK; 2 Department of Public Health and Clinical Medicine, Umeå University, SE-901 87 Umeå, Sweden; 3 Innlandet Hospital Trust, 2312 Ottestad, Norway; 4 Center for Research on Extremism, University of Oslo, 0317 Oslo, Norway; 5 St George’s Medical School, University of London, London SW17 0RE, UK; 6 School of Clinical Medicine, University of Cambridge Medical Library, Cambridge CB2 0SP, UK

**Keywords:** radicalisation, social media, public mental health, online radicalisation

## Abstract

This systematic review seeks to position online radicalisation within whole system frameworks incorporating individual, family, community and wider structural influences whilst reporting evidence of public mental health approaches for individuals engaging in radical online content. Methods: the authors searched Medline (via Ovid), PsycInfo (via Ebscohost) and Web of Science (Core Collection) with the use of Boolean operators across “extremism”, “online content” and “intervention”. Results: Following full-text assessments, all retrieved papers were excluded. No publications fulfilled the primary objective of reporting public mental health interventions specifically addressing online radicalisation. However, six publications fulfilled the secondary objective of identifying theoretical and conceptual relationships amongst elements in the three inclusion criteria (online extremism, psychological outcomes and intervention strategy) that could inform interventions within public mental health frameworks. These publications were quality assessed and discussed following the Cochrane Effective Practice and Organisation of Care guide for reporting empty reviews. Conclusions: there is an immediate need for further research in this field given the increase in different factions of radicalised beliefs resulting from online, particularly social media, usage.

## 1. Introduction

The existence and efficacy of a public mental health approach to online radicalisation is not clear. Notwithstanding debates regarding whether there is a relationship between psychological health and radicalised beliefs [1,2], as well as concerns with the use of mental health services in counter-terrorism policy [3,4,5], the number of initiatives that aim to address mental health problems or conditions as a form of counter-terrorism continues to grow. These include risk assessment protocols for vulnerable individuals [3], mental health referrals within the UK [6], and counselling and family therapy approaches in de-radicalisation [7]. Whether such approaches are effective in the long term is not currently known [4,5]. Public mental health promotion, as part of a general public health framework, by design exists outside of counter-terrorism programmes [8]. It incorporates whole-system frameworks with attention to health promotion (including wellbeing and resilience), prevention and intervention, incorporating individual, family, community and wider structural influences [8,9].

On an individual level, wellbeing can be influenced by sociodemographic factors (such as income, housing and employment), physical and psychological health, resilience, identity factors, adverse childhood experiences and/or trauma, amongst other risk/protective factors [8]. On a structural level, wellbeing can be influenced by societal discrimination, social and cultural norms, economic conditions, inequality, political structures, global politics and/or migration, amongst a number of other risk/protective factors [8]. Radicalisation depends on complex interactions amongst different risk factors—a disruption in one or several of these can lead to a multitude of negative psychosocial outcomes, such as an extreme dissatisfaction with authority, an unmet need to belong, a propensity to favour reactionary populist policy, and/or joining an extreme organisation [4,9,10].

User behaviour (whether due to psychographic profiling, users’ own interests, platform algorithms or other behaviour change mechanisms) within peer-to-peer gaming, streaming, video sharing and social media platforms has altered the landscape [11]. Radical groups that are active online include (but are not limited to) far-right, far-left, Islamist and other religious groups, conspiracy theory, “incel”, “eco-activist” and those incorporating elements from populist agendas appearing within mainstream politics [12,13,14,15,16]. On TikTok (a collaborative, short-form video hosting service), it has been noted that: 

“After watching a thousand videos [around seventeen viewing hours], recommendations [by the platform to users] become increasingly radical in nature, in content, and in tone, ultimately sending [an individual] into conspiratorial echo chambers... Mainstream conservative political material [turns] toward antivaxxer material, hypermasculinity... Hatescape, a conspiracy rabbit hole, socialization and education on hate or dissidence, and even some calls for and demonstration of violence” [17]. 

Additionally, a number of social media, video hosting and peer-to-peer networks have been criticised for contributing to psychological effects such as addiction, attention deficiency and individual and group attitudinal shift and triggered debate about inappropriate content, misinformation, disinformation, moderation, user privacy and censorship [18]. 

The six publications discussed below provide explicit and general examples of online violent extremist content (e.g., videos of violence used to achieve political aims) playing a role in radicalisation processes and suggest relationships amongst violent extremism, resilience and wellbeing. Yet, the question remains as to whether any public mental health approaches have sought to intervene in this area. Public health with attention to a public mental health foundation already is an identified framework for addressing violent extremism and radicalisation [9,19,20]. And it is important to note that there are a variety of public health frameworks, and which framework is chosen will shape the orientation in different ways with different consequences, for example, whether attention is given to public mental health *and* to public mental health promotion [21]. However, such public health frameworks have not specifically included an online focus.

The aim of this review, therefore, was to find public mental health approaches that have been utilised to identify, prevent or address online ideological and political radicalisation. In addition, this review sought to identify the nature of online content, the demographics of those accessing content and wider correlates between radicalised beliefs and factors outside of the online sphere, such as those used within public mental health frameworks. This review then aimed to frame these findings within the four working hypotheses detailed below. Broad definitions of both radicalisation and public mental health promotion were used, considering the range of very different understandings associated with both terms and the very varied consequences of such understandings. 

To encompass this range of understandings, “radicalisation” was defined using search terms that acknowledged the definitional debate around belief versus behaviour, process versus outcome, and critical scholarship. The latter identifies a research “selection bias” that perpetuates a “conceptual backformation” of radicalisation purely as a precursor to terrorism detached from the possibility of critical but nonviolent direct political action [22]. Accordingly, the search terms included “radical” separate from, as well as linked to, extremism and terrorism and also encompassed far-right, far-left, conspiratorial, Islamist and other views that have the intent of shifting the status quo. Whilst acknowledging that radicalisation only infrequently leads to violence, this review aimed to identify intervention approaches that addressed potential triggers for that outcome. “Public mental health promotion” was defined as incorporating community, school-based and clinical approaches to promote resilience and wellbeing, where one of the desired outcomes fell within the parameters of a psychological domain, building on a public mental health promotion orientation for public mental health interventions [20,23,24]. 

This review was conducted as part of the European Union’s Horizon 2020 research and innovation programme (The DRIVE project: Determining multi-levelled causes and testing intervention designs to reduce radicalisation, extremism and political violence in north-western Europe through social inclusion, see ‘Funding’ section below). It expresses exclusively the authors’ views, not necessarily those of all DRIVE project Consortium members, and neither the European Commission nor the Research Executive Agency is responsible for any of the information it contains.) focusing on social exclusion and marginalisation as experiential factors in people inclining or turning toward radicalisation (as understood above), broken down into four public mental health working hypotheses. The focus on reciprocal radicalisation between far-right and Islamist groups in the fourth hypothesis was based on reported evidence from before the COVID-19 pandemic [25], included in the project proposal submitted 19th March and awarded funding in July 2020. However, during and following COVID, the radicalisation and extremist landscape became more complex with, for example, vaccine hesitancy and surrounding conspiracy theories uniting Black and ethnic minority, highly religious groups, far-right and far-left political groups [26]. Other groups have become more prominent, as referenced above, such as “incels”, whose adherents span right, left and jihadist ideologies [27]. The following applies the DRIVE project’s public mental health set of four hypotheses to the focus of this review, online engagement. These hypotheses emerged after an initial analysis of DRIVE project interview data from four European countries focusing on young persons’ narratives relating to their thoughts and experiences of social exclusion, marginalisation, extremism and radicalisation. In reviewing this data, two central themes emerged of relevance for this review: first, knowledge of and engagement with online sites promoting radicalisation emerged as a consistent and dominant theme of concern for and by participants; second, hybrid patterns of online/offline radicalisation exposure were identified, often related to lock-down during the COVID-19 pandemic [28,29,30].

The first hypothesis concerns spatial formations, stating that a sense of safety and security, and a sense of belonging, or the lack thereof, during online engagement can operate as protective or risk factors for extremism. The second hypothesis concerns identity politics, stating that identity-building behaviours and strategies including ritualised activities, the identification of existential and everyday meaning-making symbols, and emotion manipulation techniques are used for marking in-group and out-group belonging and function to reinforce the identity process by those creating and maintaining online sites. The third hypothesis concerns intergenerational change and continuity, stating that identity and belonging are reinforced during online engagement by the attempt to reconfigure, weaken or replace existing nuclear family/clan bonds (where they exist), as well as targeting those lacking such bonds, in order to create new family/clan constellations. The fourth hypothesis concerns reciprocal radicalisation, stating that a political shift of governance can bring about new patterns of reciprocal radicalisation (e.g., related to changes in legislation and policies) that inform online content and can adversely affect health (mental health). Framed within these four hypotheses, the search strategy, corresponding results and discussion are set out below. 

## 2. Materials and Methods

**Figure 1:**
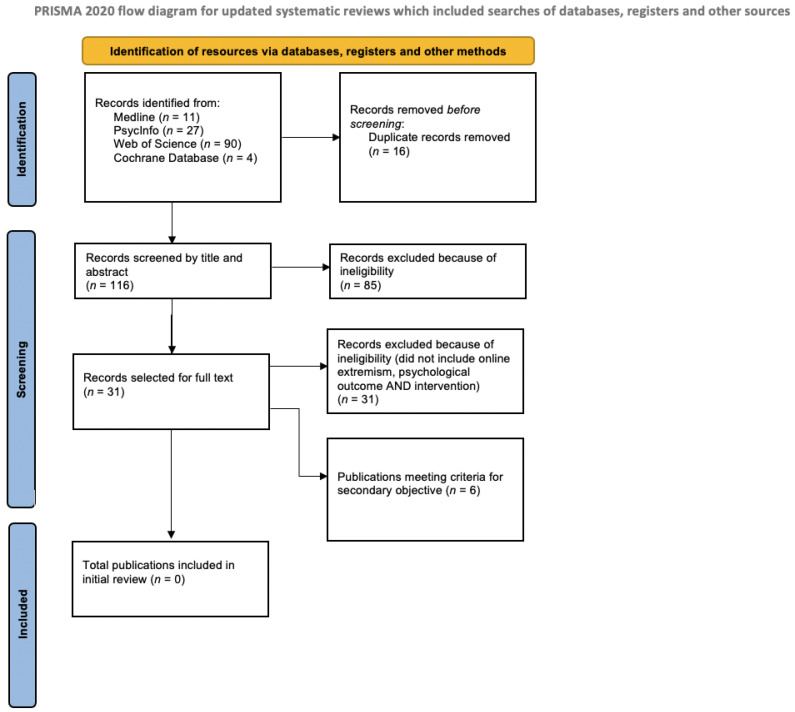
PRISMA diagram.

The search for publications included Medline (via Ovid), PsycInfo (via Ebscohost) and Web of Science (Core Collection), with the use of Boolean operators and search terms as listed in Table 1. This specific combination of databases has been shown to perform best at achieving efficient and adequate coverage of studies, and it was expected that together they would provide the best coverage of journals, topics and evidenced-based methods, to match the interdisciplinary nature of this systematic review [31]. Medline is a medical and healthcare database covering this topic from a public health perspective; PsycInfo is a psychology database covering this topic from a psychological perspective; and Web of Science provides a multidisciplinary science database which additionally covers this topic from a sociological perspective. Finally, the Cochrane Library was searched (see search terms below). The search resulted in a database of 132 publications, which was deduplicated using the Rayyan.ai systematic review screening platform. The publications were then systematically filtered in line with the eligibility and exclusion criteria outlined below. The review followed the guidelines set out by PRISMA [32]. The PRISMA flowchart (Figure 1) summarises the filtering process to exclude publications that did not fulfil the eligibility criteria. Publications were first excluded by title and then abstract, with the remaining excluded through full text. At each point of the exclusion process, the publications were assessed against the eligibility criteria. If the authors determined that a publication failed to meet the eligibility criteria, it was excluded.

### 2.1. Eligibility Criteria

Publications were limited to those that appeared within scholarly databases, including reviews, meta-analyses, narrative reviews, pre–post studies, case studies, interventions, cohort studies, cross-sectional studies, policy and public service-related work. Publications were included if they contained three elements: *firstly*, online extremist content; *secondly*, mental health and psychological processes as outcomes or variables within their focus; *thirdly*, an intervention with a clear discussion of evidence-based references. We defined an intervention study as one in which the researcher “actively interferes with nature—by performing an intervention in some or all study participants—to determine the effect of exposure to the intervention on the natural course of events” [33]. Intervention was interpreted broadly here (see Section 2.2. (Population, Intervention, Control, Outcome (PICO) criteria) below). Publications were included if they were written within the last ten years to account for the rise of social media platforms and extremist content. 

### 2.2. Population, Intervention, Control, Outcome (PICO)

The PICO framework [34] was used to address the public mental health-based enquiry arising from this review as outlined below: 

*Population*: separated by age (young: 11–17-year-olds; young adult: 18–25-year-olds; and adults: 25+); separated by milieu (e.g., Islamist, far-right, and other); 

*Intervention*: public mental health, including care in the community, child and adolescent mental health services (CAMHS) and CAMHS-like organisations across different countries, education, social service, public service partnerships, primary care, referral pathways, clinical programmes, health promotion and prevention including school-based interventions;

*Control*: publications sorted into whether or not a control group was used, to assess the clinical quality of interventions;

*Outcomes*: diagnostic outcomes, cognitive, mental health, wellbeing, resilience, psychosocial, social determinants of mental health, learning difficulties, neurodevelopmental conditions, affect and emotional responses. 

## 3. Results

### 3.1. Data Extraction and Analysis

After duplicates were removed, publications were subjected to a two-stage screening procedure. In the first stage, publications were screened by title and abstract by two authors (RM and EBM). In the second stage, publications were screened through the full text by two authors (RM and EBM) and discussed amongst the five authors (RM, EBM, VDM, MN, HL). A data extraction table was then created outlining the PICO variables, details of the interventions and each author, journal, year and other publication details. The authors independently retrieved thirty-one relevant publications (from the initial 132 publications) according to the inclusion criteria by title and abstract screening (see Figure 1). Following the assessment of the full text, all retrieved publications were excluded based on the inclusion criteria. 

No publications were identified that presented public mental health intervention approaches to online extremism, as was the focus of the initial review (see Figure 1). The primary objective of this review was to identify public mental health interventions specific to individuals engaging in online radicalisation. Since the search yielded no results, a subset of six publications were identified that addressed a secondary objective: theoretical, anecdotal or conceptual discussion of online behaviour and psychological outcomes with mention of strategies that could be used to inform intervention designs within the parameters of public mental health frameworks. This was undertaken in accordance with the Cochrane Effective Practice and Organisation of Care guide to reporting empty reviews [34]. The other 25 publications of the 31 selected for full-text review did not include elements from each of the three eligibility criteria and were excluded. The search was for publications that appeared in scholarly databases, and the six publications addressing the secondary objective, presented below, include two quantitative studies, a qualitative retrospective single case study, a book chapter (reviewed by the three editors of the volume), a peer-reviewed conference paper and an editorial. Considering the need for attention to the development of interventions to address online radicalisation, after reporting zero results, we analysed the six publications addressing the secondary objective to move forward the development of public mental health promotion approaches to violent extremism involving online radicalisation. Table 2 provides a summary of the analysed publications before the themed outline.

### 3.2. Quality Assessment 

No studies fit the eligibility criteria for the primary objective of this review. An evaluation methodology for qualitative [35] and quantitative [36] research was used to assess the quality of the peer-reviewed articles chosen for discussion. These quality assessment criteria address epistemological positioning, bias, validity and generalisability, author reflexivity and comprehensiveness of approach. For the secondary objective, authors used Stroup et al. [37] to synthesise an analysis of the six relevant publications using background (country of origin and type of publication), summary, methodological summary, central finding/argument, and reasons for primary object exclusion/secondary object relevance/quality assessment outcome. Two different tools were used for quality assessment, one for qualitative and one for quantitative research [35,36]. Neither produces a score but asks researchers to consider the presence or absence of research processes or outcomes, such as epistemological position, bias, design, validity and generalisability of the results (see Appendix A). The synthesis (following Stroup et al. as described above), and a notation concerning the quality of the assessment outcomes for four of the six appear in Table 2 below. No quality assessment was conducted for the book chapter or editorial). The six publications are discussed further in Section 3.3, Section 3.4 and Section 3.5 below.

The literature summarised above is presented below in a three-themed outline: range of online content; who accesses the content; and radicalisation and its correlates.

**Table 2:** Synthesis of relevant studies.

Name	Country/ *Milieu*	Type of Publication	Summary	Methodology	Central Finding/Argument	Reasons for Primary Objective Exclusion/ Secondary Objective Relevance/ Quality Assessment (QA) See Appendix A
Schmitt et al., 2018 [38]	German/ *Islamist,* USA/ *Far-right*	Information network analysis; peer-reviewed journal article	An online information network analysis of the links between online extremist content and counter extremist messages given that the quantity of extremist messages vastly outnumbers counter messages, both use similar keywords and automated algorithms may bundle the two types of messages together: counter messages closely or even directly link to extremist content.	The authors used an information online network analysis to explore what might hinder a successful intervention addressing online radicalisation. Videos from each campaign (8 from counter Islamist, 4 from counter far-right) were listed and treated as “seeds” for data collection using the online tool *YTDT Video Network.* For each list of “seeds” related videos and metadata were retrieved, including view count, rating and crawl depth to 2. To reduce biased results due to the researchers’ own search history, browser history and cookies were deleted before retrieving the data.	Extremist messages were only two clicks away from counter messaging. Aiming to integrate the role of recommendation algorithms into the “selective exposure” paradigm, the authors suggest that the algorithm filtering and gatekeeping functions directing content and users toward one another, including user amplification through sharing and “likes”, as well as the overwhelmingly larger volume of online extremist compared to counter extremist content, together pose almost insurmountable challenges to online interventions targeting or countering extremist content.	Excluded because of nonreporting of the testing of an intervention. Relevance: the article addressed online extremist content, its relationship with user behaviour and attitudinal shift and analyses of interventions used. Public mental health approaches might utilise online counter messages as part of an intervention and several obstacles to counter messaging efficacy were identified. QA: all components were present.
Rusnalasari et al., 2018 [39]	Indonesia/ *Islamist*	Cross-sectional analysis; peer-reviewed conference paper	An analysis of the relationships amongst literacy and belief in and practice of the Indonesian national ideology of Pancasila and literacy in extremist ideological language, with the view of demonstrating that belief and literacy correlate with reduced vulnerability to online radicalising content; belief and literacy were negatively correlated with vulnerability.	The authors developed and reliability tested two new measures and then used them to explore if high literacy in a national ideology coupled with literacy regarding language used in extreme ideologies decreased vulnerability to (or offered protection against) online radicalisation.	High levels of literacy in national ideology and extremist ideologies was not found to reduce vulnerability to or offer protection against online radicalisation.	Excluded because of not reporting the testing of an intervention. Relevance: the paper addressed online extremist content, its relationship with language outcomes in the cognitive domain and theorised the type of intervention that may be useful within education settings QA: some components were present.
Bouzar and Laurent, 2019 [40]	France/ *Islamist*	Retrospective single-case study analysis; peer-reviewed journal article	A qualitative interdisciplinary analysis of the radicalisation of and disengagement intervention with “Hamza”, a 15-year-old French citizen who attempted several times to leave the country to prepare an attack on France; an analysis concluded that Hamza’s life course and related trauma experiences led to radicalisation through the interaction of 3 cumulative processes: emotional, relational and cognitive–ideological.	The authors retrospectively reported on the use of inter-disciplinary (psychological, social, political and religious) thematic analyses of semi-directive interviews and online communications with extremist recruiters to identify the radicalisation stages that led to a young person’s attempts to commit extremist violence and the conditions necessary for a successful intervention.	Based on the successful outcome of this case study, it was argued for the efficacy of a multidisciplinary intervention that analyses an individual’s life trajectory (rather than only one or two time points) informed by two first steps: (i) thematic analyses of semi-structured interviews with parents and the radicalised individual; (ii) when permission is granted and access is legal, thematic analyses of mobile phone and computer records revealing the frequency, content and patterns of engagement between the individual and the extremist recruiters.	Excluded because of not describing in detail the intervention methodology beyond the initial steps. Relevance: the article addressed online extremist content, its relationship with several psychological domains including affect related trauma and outlined the outcome of the first steps of an intervention. QA: several components were present implicitly and a few were absent.
Siegel et al., 2019 [41]	Global/ *Several*	Narrative review; book chapter; chapter proposal and subsequent drafts were reviewed by three editors who had to agree that a draft met quality and relevance criteria for the book	A book chapter reviewing pathways to and risk factors for radicalisation, theoretical explanations as to why youth may become radicalised and recommended intervention approaches and examples in six overlapping arenas (family, school, prison, community, internet and government); review concludes that trauma-informed approaches across the six interacting systems are required.	The authors offered a chapter-length overview of reducing terrorism and preventing radicalisation in six overlapping arenas: family, school, prison, community, internet and government (the latter referring to diverse services at the international, national and local levels, depending on the country and region, e.g., resource provision to schools, prisoner aftercare, public–private partnerships, financial support services, internet monitoring and law enforcement).	Identified five arenas overlapping with the digital arena in which interventions should be located (family, school, prison, community and government) and argued that two needed approaches are largely absent: trauma informed and resilience promotion.	Excluded because of the absence of the reported testing of the intervention; specific interventions simply mentioned as examples Relevance: the chapter addressed online extremist content, its relationship to trauma and theoretical areas where interventions may take place. QA: not applicable.
Tremblay, 2020 [42]	Global/ *Extreme right-wing, far-right*	Narrative review; nonpeer-reviewed editorial	An editorial focussing on the alt-right movement, using the terrorist attacks in Christchurch in 2019 as an example: the attack was “A sign of our digital era and social-mediatized gaze”, having been live streamed on Facebook and widely shared across the virtual community. The development of inclusive habitats, governance, systems and processes were identified as significant goals for health promotion to foster “peaceful, just and inclusive societies which are free from fear, racism, violation and other violence”.	The author provided a very brief high-level analysis focusing on the intersectionality of discrimination and oppression with radicalisation in the digital, political and social spheres.	Argued for multisector partnerships with public mental health promotion approaches to reduce discrimination, oppression and radicalisation in the digital, political and social spheres.	Excluded because of the absence of the reported testing of the relevant interventions. Relevance: the editorial addressed online extremist content and areas within public mental health promotion where interventions may take place. QA: not applicable.
Schmitt et al., 2021	Germany/ *Anti-refugee*	Between-subjects design; peer-reviewed journal article	The first study examining the effects of two different narrative structures, one-sided (counter only) or two-sided (extremist and counter) using the persuasion technique of narrative involvement operationalised as two different types of protagonists (approachable or distant/neutral). The narrative focused on a controversial topic (how to deal with the number of refugees in Germany) and the effect of each narrative structure on attitude change was measured; participants who read the two-sided narrative showed less reactance; the smaller the reactance, the more they felt involved in the narrative which, in turn, led to more positive attitudes towards refugees; variations in the protagonist failed to show an effect.	The authors drew on findings from the earlier Schmitt et al. (2018) [39] study, theoretical concepts and studies around one-sided versus two-sided narratives, and narrative involvement, to examine the factors involved in persuasiveness. Measured manipulations (one- and two-sided narratives; identification with protagonist), attitude change, freedom threat and narrative involvement. No control groups, follow-up or behavioural change measures.	Reported less reactance from a two-sided versus one-sided narrative, that is, from a narrative that included an extremist as well as a counter message. Less reactance was accompanied by increasing narrative involvement (measured as transportation into the narrative and identification with the main character) and self-reported positive attitudinal change toward refugees.	Excluded because of the absence of the reported testing of an intervention. Relevance: the article reported a study that could inform an intervention design using counter messaging. Addressed online extremist content, its relationship with user behaviour and attitudinal shift and analyses of the psychological mechanisms involved in mediating the effects of different narrative structures. QA: Most of the components were present.

### 3.3. Range of Online Content

The six publications summarised above (three peer-reviewed journal articles, a peer-reviewed conference paper, a book chapter reviewed by the three editors of the volume, and an editorial) outlined a range of online content, mainly shared within social media and video platforms such as YouTube. Analysis of extremist online content varied across the publications, from granular assessment of narrative structure to words associated with terrorism to general descriptions or official definitions, as well as content targeting users’ existential questions and psychosocial needs. Importantly, all publications within this search noted the interaction or fluidity between the online and offline worlds, with external influences, such as education levels or socioeconomic factors, most strongly impacting cognitive and behavioural outcomes.

#### Type of Online Content across Publications

Schmitt et al. [38] conducted an information network analysis to determine the likelihood that users viewing counter extremist videos would also access extremist informational and commentary style videos. They defined online extremist content as that involving a desire to impose an alternative ideology radically, forcefully or violently, with totalistic claims stemming from what is considered to be a “true understanding” of the world (p. 782). Counter extremist content involved alternative “positive” or “civic education” content with the purpose of steering viewers away from extreme content, such as hate speech, conspiracy theory or propaganda (p. 783). The analysis in this study examined two “exemplary” counter-messaging campaigns: #WhatIS, an anti-Islamist platform run by the German Federal Agency of Civic Education, and Life After Hate, an anti-far-right platform run by ExitUSA. The authors cited studies indicating that the efficacy of counter messaging was mixed, and persuasive mainly with those already expressing doubts (p. 783). They went on to describe how the effectiveness of online counter messaging is reduced further by the combination of similar keywords being used by both counter and extremist sites, the organising and gatekeeping functions of algorithms that direct content and users toward one another and the much greater volume of extremist content that exists on platforms (pp. 784–786). 

Rusnalasari et al. [39] proposed that “vulnerable” internet users, particularly adolescents, access online content pertaining to “individualism, fundamentalism, radicalism and terrorism” (p. 1). Noting that online radicalisation was one of the contributing factors to the Bali bombings of 2002, the authors asserted that increased understanding of online radicalisation processes could address or prevent future instances of violent extremism. Bouzar and Laurent [40] also focused on the interaction between online content and the needs of internet users. They presented a single-subject case study of fifteen-year-old “Hamza”, in France, whose unresolved mourning, sense of societal injustice and existential questioning were exploited by online ISIS recruiters until he desired martyrdom as entry into a new life. Noting that most ISIS recruits, aged 15–30, are looking for an idea, a group and strong emotions, the authors analysed the recruiters’ messaging history with Hamza and identified complex emotional and relational strategies that effected cognitive change to achieve group membership, loyalty and self-sacrifice. The authors presented a calculated, interactive model of online engagement by ISIS recruiters with increasingly extremist online content accessed through and accompanied by frequent communication with recruiters on social media. The conclusion was that a “perfect storm” occurred involving a vulnerable young person interacting with online recruiters who provided answers to existential questions that family members, school friends and teachers, community and religious leaders, and wider social online and offline systems failed to address. 

Siegel et al. [41] reviewed theoretical explanations of radicalisation using a trauma-informed perspective to examine risk factors. They identified overlapping factors in family, school, prison, community, governmental (e.g., resource provision to schools, prisoner aftercare, public–private partnerships, financial support services, internet monitoring and law enforcement) and internet environments that together contribute to radicalisation in different countries (e.g., US, Europe and Australia), rather than simply the content of online material alone. Whilst careful not to attribute causality to trauma, the authors theorised that trauma experiences not met with trauma-informed support, combined with family, community, public institutional and governmental policy factors that interacted with online extremist content, could lead to radicalised beliefs and actions. 

Similarly, Tremblay [42] argued that a “vicious interplay” between digital, societal and political spheres contributes to radicalisation and that public mental health promotion “ought to play a role in addressing the factors contributing to extremism” (p. 2). Tremblay in addition cited various public, institutional and social factors that can promote current trends in extreme right-wing ideology. 

Schmitt et al. [43] examined whether online content containing characters that the reader can identify and empathise with (i.e., are approachable rather than distant and neutral) was more conducive to cognitive manipulation. The authors focussed on narratives around immigration, refugees and socioeconomic divides, counter messages directed against extremist ideologies and violent tendencies that exposed the manipulative or propagandic nature of extremist messages. Considering narrative engagement as determinative of how profoundly the content impacts the user, they argued that through narrative involvement a user may temporarily lose connection to reality and escape into the character’s world. In other words, the more users are transported into a story, the more likely they are to engage in story-consistent beliefs and be susceptible to persuasion, which, in turn, can deepen or counter radicalised beliefs, depending on the content [43].

### 3.4. Who Accesses Online Content

All six of the publications discussed within this secondary objective review assumed that online content is widely and increasingly accessed across the general population and acknowledged extensive use by those exploring ideas, looking for answers, and already involved in some way or committed to extremist groups. Whilst noting that extremist online content can target young users, the publications examined “who” by considering why and how users engaged with extremist or counter extremist content and the attitudinal and/or behavioural effects of that engagement. 

#### Target Populations across the Publications

Schmitt et al.’s information network analysis of extremist and counter extremist messaging on YouTube identified behaviours particular to internet users who engage in hate speech, propaganda, violent extremism or conspiracy theories [38]. For example, to spread beliefs, users predominantly rely on social media channels, where messages and ideas can reach a wider audience at a faster rate than in person and then filter through networks of social media users. The authors reported that messages often target younger users through popular media culture, such as gaming, music videos and viral videos, frequently using “wolf in sheep’s clothing” tactics [38] (p. 783). Rusnalasari et al. examined the effectiveness of a form of civic education in equipping young people to recognise and turn away from extremist online content [39]. This cross-sectional study used data from 193 13–21-year-old participants recruited using purposive sampling through social media links and local contacts who self-reported as active internet users. Extremist ideological literacy levels were correlated with belief in a civic ideology, *Pancasila*, the Indonesian national ideology of peaceful co-existence amongst five religions: Islam, Confucianism, Catholicism, Protestantism and Buddhism. However, the authors found little evidence of protection against radicalisation from the combination of belief in *Pancasila* and literacy in the extremist ideological language used online to promote terrorism. Similarly, a case study by Bouzar and Laurent [24] plotted the psychosocial journey of a 15-year-old French citizen who accessed not only online extremist content during an existential crisis but interacted online with ISIS recruiters, who subsequently convinced him to leave France for Syria. Schmitt et al.’s 2021 analysis of an older group of 405 participants (mean age: 40.68, SD = 15.15, recruited via a nonprobability access panel or “convenience pool”) found reduced reactance from a two-sided versus one-sided narrative, that is, from a narrative that included an extremist as well as a counter message [43]. Reduced reactance was accompanied by increasing narrative involvement (measured as transportation into the narrative and identification with the main character) and self-reported positive attitudinal change toward refugees. Siegel et al. offered a theoretical outline of possible pathways to radicalisation for young internet users, noting that those who have experienced trauma within various psychosocial events are at higher risk of radicalisation [41]. Tremblay [42] asserted that far-right terrorist events and hate crimes evidence a deeper social and wellbeing malaise played out in the overlapping digital, societal and political spheres, citing work on the relationship between discrimination and health inequalities by Krieger [44] and the World Health Organization [45]. Racism and discrimination expressed and experienced across all three spheres can create and reinforce ideas of social dominance and oppression. Tremblay [42] noted that associations have been found amongst social experiences of discrimination or oppression, impaired biological function and reduced capacities to adapt and cope with social and contextual challenges. Tremblay also cited arguments by Wilkinson and Pickett [46]: that as inequity increases, violence and “perceived threat to pride” increase which, in turn, drives radical and extremist narratives and reinforces maladaptive reactions, such as oppressive and discriminatory behaviours (p. 2). For populations who experience social malaise, Tremblay noted, online content can reinforce radicalised behaviours.

### 3.5. Online Radicalisation and System-Wide Frameworks

The six publications that were relevant each approached radicalisation from different angles and, therefore, identified a range of correlates that varied in type and kind: the function and role of social media algorithms; the combination of two types of literacy, in a national ideology *and* in words that can be encountered online that are associated with terrorism; online recruitment discourses targeting psychosocial needs; trauma events and experiences in a world with decentralised, ever more accessible internet and social media; public mental health promotion research, oppression and the role of the digital sphere; and the narrative structure of online counter messaging. An increasingly hybrid world where lived realities occur simultaneously online and offline involves a wide array of correlates associated with radicalisation, criss-crossing sectors, domains and conceptual frameworks. 

#### Interactions between Extremist Content and the Online/Offline Space

Schmitt et al.’s 2018 article reported on algorithmic interconnections between counter extremist videos and extremist videos in two “successful” campaigns that posted counter-messaging videos on YouTube [38]. In doing so, the authors examined the way users can interact with the videos in each campaign. The study showed that extremist content could lead to counter extremist content if relevant keywords were used within counter extremist content. However, even if the keywords differed, extremist content online could still be accessed within two clicks of a counter message. In contrast, it was unlikely that viewers of counter-messaging content would view more *counter* content given the personalisation algorithms that direct similar content toward users, the overwhelmingly larger number of online extremist messages compared with counter messages and the “relevance algorithms” that focus on activity level rather than popularity metrics (p. 798).

To examine the interactions amongst literacy and belief in a national civic ideology (*Pancasila*), literacy in extremist ideological language and vulnerability to extremist recruiters, Rusnalasari et al. [39] used a cross-sectional design with an online survey to collect responses to (i) nineteen questions measuring literacy concerning extreme words that could be used to promote terrorist ideologies (3–5 items per concept—individualism, conservatism, fundamentalism, radicalism and terrorism); (ii) five questions measuring participants’ understanding of the civic ideology (*Pancasila*); and (iii) “a few questions” collecting demographic information (p. 3). Prior to correlation analyses, the questionnaires were validated and reliability tested using logical regression with a finding of 95% confidence levels (p. 3). The authors found that higher literacy levels concerning extreme ideologies were interconnected and correlated with higher understandings of *Pancasila*. Despite this finding, “several” further questions regarding how to put into practice the belief of peaceful co-existence elicited 70% “wrong” answers, which the authors hypothesised indicated being “vulnerable” to “decide the wrong reaction” that would tend to “change into action of terrorism” (p. 5). This was not explained or explored further in the publication, and no other publication in English was found that reported on this research. 

Bouzar and Laurent [40] conducted qualitative thematic analyses of semi-directive interviews with the subject, “Hamza”, and his parents. With permission from the parents and Hamza, and with legal approval, the interdisciplinary disengagement team conducted an analysis of Hamza’s online engagement with ISIS on his computer and mobile phone. This represented unusual access to the videos viewed by an individual and shared by recruiters within the IS group during a process that led to violent extremism. These first steps of the intervention (interviews and analysis of online engagement) produced combined analyses that revealed the emotional, relational, psychological and social stages and reasoning processes underlying a targeted individual’s radicalisation process that began online. The subsequent steps of the intervention continued with this multidisciplinary approach but were not described in detail and only referred to generally as part of continued engagement for a period of time. The article focused on the benefits of and need for an interdisciplinary approach using thematic analysis and, where ethically viable, access to mobile phone and computer records to analyse engagement with recruiters as part of an intervention that will “untie” violent extremist beliefs [40] (p. 664). As researchers and practitioners, the authors emphasised that to be authoritative the extremist recruitment discourse makes a difference in the young person’s life and, therefore, the disengagement discourses must *also* make a difference in the young person’s life. Methodologically, the disengagement intervention steps were depicted as targeting the explicit discourse of the online recruitment process and the implicit motivations of the young person that were identified during the thematic analyses. 

Siegel et al. [41] did not report on a specific intervention but recommended intervention parameters. Reviewing theoretical explanations of adolescent and young adult radicalisation, they used a trauma-informed perspective to examine the risk factors for radicalisation. They identified family, schools, prison, community, internet and government programmes and services (for example, counter terrorism strategies) as six inter-relating arenas of radicalisation and, therefore, entry points for intervention. Radicalisation was defined as the process of adopting an extremist belief system, including “the willingness to use, support or facilitate violence, as a method to effect societal change” (p. 392, quoting Allen [47] (p. 4)). Their focus was particularly on radicalisation as a precursor to terrorist activities, understood as “any action intended to cause death or serious bodily harm to civilians or noncombatants with the purpose of intimidating a population or compelling a government or international organisation to do or abstain from an act” (p. 392, quoting the United Nations [48]). Whilst acknowledging that no clear profile or universal list of risk factors for radicalisation or terrorism exists, Siegel et al. asserted that the examination of the radicalisation process often reveals trauma events and experiences, as well as a blurry border between widely held ideologies and radicalised belief systems that can lead to violent acts. They observed that most terrorist organisations employ youths aged 15–22 years old in some capacity, recruiting by offering a clear identity, belonging and adventure. Offering an overview of the many theories about why some move from cognitive to violent radicalisation, they commented that none directly touch on the role of trauma in the radicalisation process and called for research on the roles of trauma in radicalisation and of resilience promotion in preventing radicalisation, including community-based public (mental) health promotion. As decentralised systems, impossible to control, censor or restrict and with ever-growing accessibility, the Internet and social media were named as the most significant resources in the radicalisation processes. Counter-messaging, incentivising reporting, increasing digital literacy and online dialogue with extremists by trained volunteer scholars were cited as example interventions [41] (pp. 408–409).

Tremblay [42] argued that public health (including mental health) promotion can support coordinated multisectoral models to empower those who are oppressed and to meet oppressors “where they are” [42] (p. 3). These efforts should include aims to develop skills in critical thinking, digital literacy and promote inclusive social norms. Tremblay concluded that public health, including mental health, promotion must prioritise research (including interventions) on the associations amongst inclusion, fairness and health within the economic, political, social and cultural landscapes. Calling for public health promotion involvement in this field, Tremblay noted that immigration is instrumentalised by extremist ideologies and nationalist agendas promulgated in digital, social and political spheres, represented, for example, by the “Great replacement theory” [42] (pp. 1–2).

Schmitt et al. [38] compared the effectiveness of two types of online “counter-messaging” narrative structures in changing user attitudes (and, theoretically, behaviour). The volume of extremist messaging vastly outnumbers counter messaging, and the latter sometimes contains extremist content in order to deconstruct it. But this can make the user vulnerable to extremist content through algorithm gatekeeping, filtering and amplification mechanisms, which can lead users to extremist content without the user realising it. Given this potential risk, the authors sought to determine if one- or two-sided narratives were more effective at changing attitudes, the former presenting only a counter message, the latter presenting both the counter message and the message being countered. The study was presented as the first of its kind, synthesising findings from several previous studies (e.g., [49,50,51,52,53]) and from psychological research used in advertising (e.g., [54,55]). The authors also examined the role of reactance, defined as “a physiological arousal in reaction to a certain external stimulus which occurs if people feel that their freedom of opinion is being threatened” [43] (p. 58). Defining freedom threat as an essential condition of reactance and an antecedent to further affective and cognitive aspects of experiencing reactance [43] (p. 58), the study argued that elevated reactance renders a reader less likely to accept a persuasive message. In contrast, less reactance supports narrative involvement and lowers the risk that a reader will feel that they are being forced toward a particular position. In this study, four alternative texts were presented to participants. Each narrative presented a description of a young woman, named Lena, who has strong positive attitudes towards refugees in Germany. Lena meets her long-term friend Anne. By chance, they start talking about the refugee crisis in Germany. As proposed by Cohen and colleagues [50], in the ease of identification condition, one character, Lena, is portrayed as more positive and virtuous, and she is described in detail, whereas these attributes are missing from the other character, Anne (without portraying her negatively). Two political opinions about refugees are presented: one character (Lena) expresses pro refugee arguments, whereas the other character (Anne) presents contra-refugee attitudes. In this condition, the two friends start to debate the topic. The arguments alternate between pro- and contra-asylum seekers. The debate becomes increasingly emotional and ends with the suggestion to talk about something else to prevent a serious fight. In contrast, the one-sided narrative presents only the pro-asylum seekers arguments by Lena, whereas Anne is a neutral audience to Lena’s arguments resulting in no emotional debate. 

## 4. Discussion

The initial planned review found no individual-, family-, community- or institutional-based public mental health intervention studies related to online extremism. Our primary objective was to investigate the nature of online extremist content, the demographics of individuals who access extremist content, and interventions focusing on psychological domains using a public mental health approach. We searched in scholarly databases for publications that included reviews, meta-analyses, narrative reviews, pre–post studies, case studies, interventions, cohort studies, cross-sectional studies and policy and public service-related work. Inclusion depended on the content containing three criteria: (i) online extremist content with a main focus on right-wing and Islamist extremism but possibly a subfocus on other forms of extremist content, e.g., anticapitalist extremist content; (ii) mental health and psychological processes; and (iii) description of an intervention using the PICO criteria. Intervention was defined as a programme in which the researcher “actively interferes with nature—by performing an intervention in some or all study participants—to determine the effect of exposure to the intervention on the natural course of events” [33] (p. 137). Searches on multiple databases found 132 publications that were double screened down to 31 publications with no publication fitting all 3 criteria. A search of the Cochrane Library for high-quality controlled trials, randomised or quasi- randomised, did not yield any registered studies. The secondary objective review included six publications of relevance to the topic but that did not fully fit the primary objective eligibility criteria. These six publications contained elements related to each of the three eligibility criteria, whilst the other 25 publications did not. The six publications are presented here to provide a sample of relevant literature addressing online extremism through varied methodologies (e.g., information network analyses and cross-sectional studies) or approaches (e.g., international overviews and editorial arguments) and linked in some way with public mental health approaches.

The studies that were relevant, whilst not completely fulfilling the original criteria, did identify challenges to addressing online radicalisation and violent extremism. Two of the studies focused on the hurdles related to designing and disseminating online counter extremist messages that are persuasive and do not amplify extremist content or inadvertently lead users to extremist content [38,43]. A third claimed to show that high literacy levels about extremist language and a nationalist ideology of peaceful coexistence do not reduce vulnerability to online extremist views, but details were not provided, and no further English-language publication was found [39]. A single-subject case study was presented to argue that, although most disengagement programmes view radicalisation processes from a single time-point and one disciplinary perspective, the emotional, relational, psychological and social tactics that recruiters use to target potential recruits with online messaging and video content require a multidisciplinary life trajectory approach to enable successful disengagement [40]. The book chapter review of the theoretical explanations of radicalisation recruitment and interventions described the Internet as decentralised; ever more accessible; impossible to control, censor or restrict; and as online spaces where help is often sought for trauma events and experiences that are embedded in many descriptions of radicalisation [41]—and, indeed, that is what Bouzar and Laurent [40] illustrate in their single case study. The editorial noted the interplay amongst digital, societal and political spheres in radicalisation and extremism, arguing for public (mental health) promotion involvement in addressing the social, economic and psychosocial factors that contribute yto experiences of oppression and oppressive behaviours toward others [42]. All highlighted the interaction amongst psychological factors, life experiences, and the wider social, economic, health, cultural and political context. In addition to being culturally informed and contextually appropriate, the findings from this review emphasise that effective evidence-based wellbeing and health promotion, prevention and intervention programmes will be part of multisector, multidisciplinary and multiagency approaches and will also provide more granular guidance for addressing online extremism. 

### 4.1. Spatial Formations

Spatial formations, as stated in the first public mental health working hypothesis presented in Section 1 (Introduction), occur in spaces where, “*a sense of safety and security, and a sense of belonging, or the lack thereof during online engagement, can operate as protective or risk factors for extremism*”. Such spatial formations are found within the online world through online groups and communities that may contribute to group-think or ideological polarisation. Analysing scraped data from a white supremacist online forum, Stormfront.org, Gregory and Piff [56] found that both cognitive complexity and style matching decreased as engagement increased, indicating increased ideological polarisation during ongoing engagement rather than the deindividuation that characterises groupthink. Studying online video games, Robinson and Whittaker [57] argue that interactive gameplay and the use of iconography such as Nazi memorabilia creates conditions of belonging that can include adherence to extremist ideologies. Whilst *spatial formation* is not mentioned specifically in any of the publications, the single-subject case study outlined in great detail the formation of identities within the spatial intersection of online and offline engagement [40]. Similarly, the editorial by Tremblay [42] noted the interplay amongst digital, societal and political spheres in which social inequalities, social determinants of health and economic, political, social and cultural landscapes create contexts of oppression, discrimination and deeper social and wellbeing malaise conducive to extremist ideologies and nationalist agendas that circulate. Additionally, different types of media and online content are used by individuals to access extremist material. The literature both within this review and otherwise (see, e.g., [17]) identified social media, peer-to-peer, video hosting and collaborative platforms as conduits to extremist material. And there appears to be a growing number of radicalised individuals amongst younger users of short-attention video platforms, such as Tik Tok, gaming platforms, including Twitch, or online videogames [56]. Tik Tok, for example, is notable for its lack of enforcement around community guidelines and has been implicated in the rise of alt-right sentiment amongst young people [17] alongside Islamist content accessed by different young populations [57,58]. 

Prolonged exposure to social media may impact long-term mental health outcomes, including stress-related, disordered-eating-related and cognitive- and attention-deficit-related outcomes [59,60], and mental health services are increasingly incorporating the long-term addictive and affect-related effects of social media into treatment plans for adolescents [59,60]. With this in mind, the question can be raised as to the purpose of interventions within this field. For the two Schmitt et al. articles ([38,43] each with the same lead author but different co-authors) that appeared within this search, the purpose of intervening through counter messaging was to affect behavioural change and persuade individuals to believe in an alternative viewpoint. Within these articles, a two-sided counter extremist narrative appeared to be more effective at persuasion rather than one-sided narratives [43], alongside facilitating identification with a main character, which transports the user into a different narrative. However, as the authors noted, even carefully designed two-sided narratives may fail to divert users from extremist content, radicalising or reducing the impact of such content because of a number of reasons. Firstly, the vastly greater quantity of online extremist content “drowns out” the “counter-voices”; secondly, counter messages need to use the same extremist wording, catchphrases or conspiracy content to attract viewers but, as a result, extremist content is only two clicks away; thirdly, counter messages accessed online globally often use humour or satire that risks being misunderstood by users in different cultures. Counter messaging, therefore, may be unproductive (e.g., reinforcing already held views) and unethical if used by policy-makers to fulfil a particular narrative. There are, as Hurlow et al. [6] point out, ethical concerns regarding “the requirement that we monitor and report all unacceptable thoughts” (p. 162). With these caveats in mind, the storytelling technique employed by Schmitt and colleagues could be employed within video content services using “nanolearning” approaches associated with superior learning retention times compared to long-form video content [61]. Moreover, counter messages like those used by Schmitt and colleagues could be used within public mental health promotion interventions. However, ethical dilemmas remain. It is not clear, for instance, who should define what constitutes a “radical” message, whether public mental health bodies should promote political attitudinal shifts or whether users or platform designers should be employed in the dissemination of counter-messaging content. 

### 4.2. Identity Politics

Identity politics, as in the second public mental health working hypothesis, involve “*identity-building behaviours and strategies including ritualised activities, the identification of existential and everyday meaning-making symbols, and emotion manipulation techniques [that] are used for marking in-group and out-group belonging and function to reinforce the identity process by those creating and maintaining online sites*”. The identity politics of exclusion, discrimination and marginalisation were mentioned explicitly within the outlined literature with a particular focus on the interaction between the online and offline worlds, including external influences such as education, community, and socioeconomic factors [41,42]. The editorial by Tremblay [42] outlined the impact of these external factors on far-right violence, with mention of the Christchurch Mosque shootings in 2019. In the single-subject case study by Bouzar and Laurent [40], Hamza’s experiences of marginalisation and discrimination within the family (for example, not being allowed to mourn the death of his grandfather) compounded with experiences of marginalisation and discrimination at the community and system levels (for example, being bullied and othered at school for being ethnically different). These experiences created a fragility within his sense of identity that was exploited by ISIS recruiters. In all of these references, strategies of ritualised activities, meaning-making symbols and emotional manipulation techniques were touched upon as part of the identity-shaping radicalisation process. As argued by Bouzar and Laurent [40], interventions effective at disrupting the radicalisation process need to be interdisciplinary and multisector to engage at the individual, family, community, and system levels. 

### 4.3. Intergenerational Change and Continuity

Intergenerational change and continuity, as in the third public mental health working hypothesis, recognised that “*identity and belonging is reinforced during online engagement by the attempt to reconfigure, weaken or replace existing nuclear family/clan bonds (where they exist) as well as targeting those lacking such bonds, in order to create new family/clan constellations*”. Intergenerational factors played explicit and important roles in the single-subject case study [40]. Even during the disengagement process, the authors reported that the parents and son never discussed how difficult it was for him to be disallowed from visiting his dying grandfather in hospital, despite their closeness, and from attending the funeral in Algeria. The parents wanted to protect him from the shock of seeing a loved one covered in tubes and machines, but the son needed to participate in the farewell and mourning rituals to go through the bereavement process and experience closure. The unresolved anger and despair felt by the son created identity and belonging fragilities, as well as existential questions about life and death. Not finding help in the local mosque, where these topics were not discussed, he turned to the Internet for answers where online IS recruiters provided answers based on a distorted version of Islam. Moreover, he came to view his family members as unfaithful, since they failed to teach him the true Muslim faith and aspired to ensure his family’s place in paradise through his own martyrdom. Intergenerational conflict is a common feature of family and community life. Public mental health approaches could benefit from utilising an ”intergenerational solidarity lens” to promote fairness and cross-generational involvement in decision making; this would engender resilience across the life course against online exploitation by violent extremist recruiters of this kind of conflict [62].

### 4.4. Reciprocal Radicalisation

Reciprocal radicalisation as stated in the fourth public mental health working hypothesis, represents “*a political shift of governance [that] can bring about new patterns of reciprocal radicalisation (e.g., related to changes in legislation and policies) that inform online content and can adversely affect health (mental health)*”. Empirical findings related to the 2015 shifting of immigration and asylum policies, for example, in the Swedish case studied in a recent Horizon 2020 project, provide an example of multilevel societal consequences brought about by restrictive policies not only for refugees, asylum seekers and their families but in the larger society as well [63]. In a short span of time, changes in labels and societal perceptions of specific immigrant groups by the majority culture and within immigrant subcultures, and from immigrant groups to the majority culture, transformed the social attitudes amongst migrant and majority groups from open and supportive to negative and adversarial. These changes were evident across fluid, hybrid online–offline spaces, creating and exacerbating intergroup tensions, particularly emerging post-COVID. This was also illustrated within the Bouzar and Laurent single-subject case study [40] in which both domestic and global politics played a role in the pathway to engaging in radicalised discourse. The authors noted that Hamza was willing to justify violent and vigilante behaviour towards French citizens because of violent and graphic content shown to him by an ISIS recruiter. Attitudinal shifts were seen when Hamza moved from wanting to help the child orphaned by the air strike to being ready to commit to killing French citizens on the child’s behalf. Similarly, Tremblay [26] noted that oppressive experiences can create oppressive behaviours. Public mental health approaches using multisector approaches at the macro-, meso- and microlevels are needed to promote mental health, resilience and wellbeing across population groups, communities, families and individuals. 

### 4.5. An Argument for the Development of the Public Mental Health Approach

The existence and efficacy of a public mental health intervention approach to online radicalisation is a topic for future research. The primary objective of the present systematic review was to search for a wide range of public mental health intervention approaches in community, education, health and other settings, but none were found that matched the inclusion criteria. 

In the revised review (i.e., secondary objective) of the six publications examined here, one publication reported on an attitudinal change study [43] that examined the psychological mechanisms involved in counter and two-sided narrative designs that could be embedded within extremist content on video hosting sites such as YouTube. Whilst the two-sided design was found to be more effective within the metrics used, the authors noted the inherent limits of online counter-messaging given the much larger volume of extremist content and algorithmic filtering and gate-keeping functions. This study could be used to inform the design of public mental health promotion interventions incorporating counter messaging, but this raises questions regarding what public mental health promotion involves and whether a whole system approach (for example, through increased access to education, employment, housing, life experiences, healthy relationships within family and community settings and greater structural and political security) could more effectively address radicalisation. Other publications described system-wide areas in which interventions could take place, such as within education [38,41], health promotion [42] and within peer-to-peer, social media and other grouping platforms [38,43]. The current review highlights a gap in this field. There is a need for further research into integrated ecosocial approaches to resilience promotion and violence prevention that focus on nested interconnections amongst individual, family, community and structural levels and include the fluidity of online and offline experiences. Such ecosocial approaches for public mental health promotion would take into consideration the complex political, social, cultural and contextual dimensions that need to be understood when planning for effective interventions [9,19,20]. These considerations guide the DRIVE project’s planning of public mental health promotion interventions.

### 4.6. Potential Biases and Errors in the Review Process

As a systematic review, the potential benefits of the review findings to various stakeholders are relevant and must be considered to avoid bias. However, no papers were found that fit the initial eligibility criteria and this review is, therefore, more distant from implementation than systematic reviews with included findings. Not finding any eligible papers to review, the purpose shifted from the primary to the secondary objective, to presenting papers that were relevant to the landscape of online radicalisation and public mental health rather than assessing the quality of existing interventions. A remaining stakeholder was the funder, the H2020 research programme, but no specific interest of the funder drove the review other than to carry it out. However, bias, both “metabias” (bias in the review) and bias in the included studies, can occur “if systematic flaws or limitations in the design, conduct, or analysis of a review distort the review results or conclusions” [64] (p. 226). The “ROBIS” tool identifies five potential areas of bias: question/inclusion criteria, search, review process, synthesis and conclusions [64] (p. 227). Strenuous efforts were made to reduce and avoid potential bias in all five areas. 

This systematic review appears to be the first, within the databases searched, to consider public mental health approaches to online radicalisation. As an emerging public mental health field, the research question and eligibility criteria were designed to retrieve as many eligible studies as possible whilst also applying appropriate restrictions. Terms were defined and reviewers adhered to the predefined objectives and eligibility criteria. A comprehensive three-step systematic search in three databases was performed plus a search of the Cochrane Library. The systematic search strategy was undertaken by a specialist librarian (VP). This minimised the risk of missing potentially relevant studies during the search process. However, the search included only English-language papers, and there may be relevant studies available in other languages, including those findable only through databases in other languages. As in most reviews, there is a risk of bias from the subjective lenses of the reviewers, and to reduce this risk the following steps were taken. The paper retrieval was performed in two phases independently by two reviewers. Any disagreement was resolved by discussion amongst the two initial reviewers and a third reviewer, who first read independently the disputed full texts, thereby minimising the risk of missing a potentially relevant primary study during the paper retrieval. No studies were found that fulfilled all of the eligibility criteria, eliminating the risk of emphasising the results on the basis of statistical significance. Five reviewers read and discussed the included papers in detail. Three reviewers drafted and six reviewers commented on and edited the draft. The specialist librarian reported the technical search details. The limitations of the included studies were noted. The reviewers are from a range of disciplinary emphases within psychology, medicine and library science, reducing the potential for one perspective or interpretation to dominate.

#### Statement of Bias and Reflexivity

As noted, this systematic review appears to be the first to consider public mental health approaches to online radicalisation. The systematic search involved three databases plus the Cochrane Library, spanning psychology, public health, including public mental health sciences, and sociology. A specialist librarian carried out a systematic search strategy to minimise the risk of missing potentially relevant studies during the search process. However, the reviewers agreed that grey literature should be the focus of a subsequent review and, therefore, potentially relevant papers in grey literature may have been missed.

Despite the above actions to avoid bias, some sources of bias remain that can characterise many reviews. The role of social media and other sources of news and commentary can influence the research areas that are given attention, the formulation of research questions, and funding decisions. Within the areas of radicalisation, online engagement and public mental health, attention is given to harm prevention and reduction rather than broad, multisector, multilevel and multidisciplinary health promotion approaches that target structural and systemic contributors to harm, such as social inequalities, discrimination and marginalisation. Perhaps this is because of the complexity, cost and long-term planning required for such approaches, resulting in the prioritisation of quicker, smaller, targeted prevention and reduction strategies [42]. There is also some overlap between promotion and prevention even though, by analogy, promoting mental health is not the same as preventing mental illness [64,65,66]. Within the context of this review, promoting social cohesion and the capacity to engage constructively with difference and disagreement is not the same as preventing and reducing online radicalisation, although there is overlap. Whatever the causes, there are underfunded and understudied research areas, and public mental health approaches to online radicalisation is one such area. This lack of research, as evidenced by the finding of zero studies that fit the eligibility criteria, renders this review more vulnerable to bias because of the lack of scholarly focus, discussion and debate.

Bias can also result from the orientation of the reviewers. All six reviewers considered how their educational background, prior assumptions and experiences might have shaped and informed their framing and interpretation of the question, application of the eligibility criteria, results and analysis. The reviewers represent those with and without clinical and medical backgrounds from different fields within and outside psychology, and the disciplinary diversity helped to mitigate against potential bias. The length of time involved in the field of radicalisation research varied across researchers from none to several years or longer, and this range of perspectives helped to mitigate against assumptions that could contribute to bias.

## 5. Conclusions

There is a paucity of data within this field. Whilst 6 out of 31 publications that underwent a full-text review were identified that had some relevance to this area (that is, contained elements relating to each of the three eligibility criteria), it appears not to be known through evidence reported in scholarly databases what public mental health approaches have been used to address online radicalisation. Although this is an emerging public mental health field, the results reported here demonstrate the need for further research in this area. Further research may utilise a realist or rapid evidence approach in which grey literature and items in the public domain may be included within the review protocol. Such literature may contain valuable discussions and other types of work that have been reported in other forms of literature and may merit reflection and stimulate further scientific research.

## Figures and Tables

**Table 1:** Search terms.

	Extremism Keywords	Online Keywords	Intervention Keywords	*n*
**Medline via OVID**	(“Radical Islam*” OR “Islamic Extrem*” OR Radicali* OR “Homegrown Terror*” OR “Homegrown Threat*” OR “Violent Extrem*” OR Jihad* OR Indoctrinat* OR Terrori* OR “White Supremacis* ^†^” OR Neo-Nazi OR “Right-wing Extrem*” OR “Left-wing Extrem*” OR “Religious Extrem*” OR Fundamentalis* OR Anti-Semitis* OR Nativis* OR Islamophob* OR Eco-terror* OR “Al Qaida-inspired” OR “ISIS-inspired” OR Anti-Capitalis*).ti,ab ^‡^. OR terrorism/	(“CYBERSPACE” OR “TELECOMMUNICATION systems” OR “INFORMATION technology “ OR “INTERNET” OR “VIRTUAL communit*” OR “ELECTRONIC discussion group*” OR “social media” OR “social networking” OR online OR bebo OR facebook OR nstagram OR linkedin OR meetup OR pinterest OR reddit OR snapchat OR tumblr OR xing OR twitter OR yelp OR youtube OR TikTok OR gab OR odysee OR telegram OR clubhouse OR BeReal OR Twitter OR WhatsApp OR WeChat OR “Sina Weibo” OR 4Chan).ti,ab. OR internet/OR social media/OR online social networking/	(“Public mental health” OR “care in the community” OR “mental health service*” OR “educational service*” OR “social service*” OR “public service partnership*” OR “primary care referral” OR “referral pathways” OR “clinical program*” OR “health promotion” OR prevention).ti,ab. OR community mental health services/OR health promotion/	**11**
**PsycInfo via Ebscohost**	TI (“Radical Islam*” OR “Islamic Extrem*” OR Radicali* OR “Homegrown Terror*” OR “Homegrown Threat*” OR “Violent Extrem*” OR Jihad* OR Indoctrinat* OR Terrori* OR “White Supremacis*” OR Neo-Nazi OR “Right-wing Extrem*” OR “Left-wing Extrem*” OR “Religious Extrem*” OR Fundamentalis* OR Anti-Semitis* OR Nativis* OR Islamophob* OR Eco-terror* OR “Al Qaida-inspired” OR “ISIS-inspired” OR Anti-Capitalis*) OR AB (“Radical Islam*” OR “Islamic Extrem*” OR Radicali* OR “Homegrown Terror*” OR “Homegrown Threat*” OR “Violent Extrem*” OR Jihad* OR In doctrinat* OR Terrori* OR “White Supremacis*” OR Neo-Nazi OR “Right-wing Extrem*” OR “Left-wing Extrem*” OR “Religious Extrem*” OR Fundamentalis* OR Anti-Semitis* OR Nativis* OR Islamophob* OR Eco-terror* OR “Al Qaida-inspired” OR “ISIS-inspired” OR Anti-Capitalis*) OR (DE “Terrorism”) OR (DE “Extremism”)	TI (“CYBERSPACE” OR “TELECOMMUNICATION systems” OR “INFORMATION technology “ OR “INTERNET” OR “VIRTUAL communit*” OR “ELECTRONIC discussion group*” OR “social media” OR “social networking” OR online OR bebo OR facebook OR nstagram OR linkedin OR meetup OR pinterest OR reddit OR snapchat OR tumblr OR xing OR twitter OR yelp OR youtube OR TikTok OR gab OR odysee OR telegram OR clubhouse OR BeReal OR Twitter OR WhatsApp OR WeChat OR “Sina Weibo” OR 4Chan) OR AB (“CYBERSPACE” OR “TELECOMMUNICATION systems” OR “INFORMATION technology “ OR “INTERNET” OR “VIRTUAL communit*” OR “ELECTRONIC discussion group*” OR “social media” OR “social networking” OR online OR bebo OR facebook OR nstagram OR linkedin OR meetup OR pinterest OR reddit OR snapchat OR tumblr OR xing OR twitter OR yelp OR youtube OR TikTok OR gab OR odysee OR telegram OR clubhouse OR BeReal OR Twitter OR WhatsApp OR WeChat OR “Sina Weibo” OR 4Chan) OR (DE “Internet”) OR (DE “Social Media”) OR (DE “Online Social Networks”)	TI (“Public mental health” OR “care in the community” OR “mental health service*” OR “educational service*” OR “social service*” OR “public service partnership*” OR “primary care referral” OR “referral pathways” OR “clinical program*” OR “health promotion” OR prevention) OR AB (“Public mental health” OR “care in the community” OR “mental health service*” OR “educational service*” OR “social service*” OR “public service partnership*” OR “primary care referral” OR “referral pathways” OR “clinical program*” OR “health promotion” OR prevention) OR DE “Public Mental Health” OR DE “Mental Health Services” OR DE “Social Services” OR DE “Health Promotion” AND DE “Prevention” OR DE “Preventive Health Services” OR DE “Preventive Mental Health Services”	**27**
**Web of Science (Core Collection)**	TS = (“Radical Islam*” OR “Islamic Extrem*” OR Radicali* OR “Homegrown Terror*” OR “Homegrown Threat*” OR “Violent Extrem*” OR Jihad* OR Indoctrinat* OR Terrori* OR “White Supremacis*” OR Neo-Nazi OR “Right-wing Extrem*” OR “Left-wing Extrem*” OR “Religious Extrem*” OR Fundamentalis* OR Anti-Semitis* OR Nativis* OR Islamophob* OR Eco-terror* OR “Al Qaida-inspired” OR “ISIS-inspired” OR Anti-Capitalis*)	TS = (“CYBERSPACE” OR “TELECOMMUNICATION systems” OR “INFORMATION technology” OR “INTERNET” OR “VIRTUAL communit*” OR “ELECTRONIC discussion group*” OR “social media” OR “social networking” OR online OR bebo OR facebook OR nstagram OR linkedin OR meetup OR pinterest OR reddit OR snapchat OR tumblr OR xing OR twitter OR yelp OR youtube OR TikTok OR gab OR odysee OR telegram OR clubhouse OR BeReal OR Twitter OR WhatsApp OR WeChat OR “Sina Weibo” OR 4Chan)	TS = (“Public mental health” OR “care in the community” OR “mental health service*” OR “educational service*” OR “social service*” OR “public service partnership*” OR “primary care referral” OR “referral pathways” OR “clinical program*” OR “health promotion” OR prevention)	**90**
**Cochrane Library**	Radical*, Extrem*, Terrorism, Neo Nazi, terror*, homegrown, jihad, indoctrin* supremacis*, right wing, left wing, religious, fundamentalis*anti-semeti*, nativis*, Islam*, Al-Qaida, ISIS, Anti-capitalis*			**4**
**Total**				**132**

^†^ Denotes truncation: words that continue after the asterisk; ^‡^ Denotes ‘Title, Abstract: i.e. words that appear within title and abstract of paper.

## Data Availability

Not applicable.

## Appendix A

Two tools were utilised to assess the quality of the qualitative [35] and quantitative [36] data within the publications outlined within this paper. Whilst neither tool produced a score, both consider the presence or absence of research processes or outcomes, such as epistemological position, bias, design, validity and generalisability of the results.

**Quality Assurance: Qualitative Data (Bouzar and Laurent** [40]**)**	
1. Epistemological position. Did the authors provide philosophical grounding for what has been done and why? e.g., post positivist; realist; interpretative; and constructivist.	Implicitly, through the insistence of a multidisciplinary approach, which inherently involved different epistemologies, i.e., social, psychological, psychoanalytical, geopolitical and religious.
2. Was bias addressed, if so, how?	Implicitly, through the need to have social, psychological, psychoanalytical, geopolitical and religious analyses (i.e., a multidisciplinary analysis) to understand the radicalisation stages that lead to a commitment to extremist violence.
3. Was it ensured that “valid” and “credible” accounts were given that reflect an accurate portrayal of the social reality? How?	Yes, with quotes from the semi-structured interviews.
4. Was there a quality assurance checklist?	Not stated.
5. Was there ongoing quality assurance, e.g., fieldnotes and field diaries.	Not stated.
6. Reflexivity of researcher’s position: was there transparency in the decisions made and the assumptions held?	Implied through the multidisciplinarity but not stated.
7. Comprehensiveness of approach: was the study systematically designed, conducted and analysed?	NA—retrospective case study.

**Quality Assurance: Quantitative Data**	**Schmitt et al.** [38]	**Rusnalasari et al.** [39]	**Schmitt et al.** [43]
Why was the study conducted (is it a clearly focused question that addresses population, intervention and outcomes?)	Yes	Yes	Yes
What type of study (does the study design match the question asked. Intervention questions are best answered with randomised controlled trials. Is it an RCT?)	Yes	Yes	Yes
What are the study characteristics (can this be answered by PICO?) Population, Intervention, Control, Outcome)	Yes	Yes	Yes
Was bias addressed? What was done to address bias?	Yes	No	No
Are the results valid? (i.e., treatment effect, *p*-value)	Yes	No	Yes
Can clear conclusions be made? (can the results be generalised?)	Within stated limitations	Cannot tell	Within stated limitations
